# Determination of Response Factors for Analytes Detected during Migration Studies, Strategy and Internal Standard Selection for Risk Minimization

**DOI:** 10.3390/molecules28155772

**Published:** 2023-07-31

**Authors:** Nikolaos Kritikos, Anna Bletsou, Christina Konstantinou, Antonios-Dionysios Neofotistos, Constantinos Kousoulos, Yannis Dotsikas

**Affiliations:** 1 QualiMetriX S.A., 579 Mesogeion Avenue, Agia Paraskevi, 15343 Athens, Greece; n.kritikos@qualimetrix.com (N.K.); a.bletsou@qualimetrix.com (A.B.); c.konstantinou@qualimetrix.com (C.K.); a.neofotistos@qualimetrix.com (A.-D.N.); 2 Laboratory of Pharmaceutical Analysis, Department of Pharmacy, School of Health Sciences, National and Kapodistrian University of Athens, 15784 Athens, Greece

**Keywords:** migration, polymeric materials, leachable and extractable species, mass spectrometry, design space, internal standard

## Abstract

Migration studies are one of the few domains of pharmaceutical analysis employing wide-scope screening methodologies. The studies involve the detection of contaminants within pharmaceutical products that arise from the interaction between the formulation and materials. Requiring both qualitative and quantitative data, the studies are conducted using Liquid Chromatography or Gas Chromatography coupled to a mass spectrometer (LC-MS and GC-MS). While mass spectrometry allows wide-scope analyte detection and identification at the very low Analytical Evaluation Threshold (AET) levels used in these studies, MS detectors are far from “universal response” detectors. Regulation brings the application of uncertainty factors into the picture to limit the risk of potential analytes detected escaping report and further evaluation; however, whether the application of a default value can cover any or all relevant applications is still debatable. The current study evaluated the response of species usually detected in migration studies, generating a suitable representative sample, analyzing said species, and creating a strategy and evaluation mechanism for acceptable classification of the detected species. Incorporating novel methodologies, i.e., Design of Experiments (DoE) for Design Space generation, the LC-MS-based methodology is also evaluated for its robustness in changes performed.

## 1. Introduction

Polymeric materials that are commonly used as containers in the pharmaceutical industry contain several additives that are used to enhance the properties of the containers. Typical classes of additives include antioxidants, plasticizers, anti-degradants, colorants or adhesives. These additives or their degradation products, as well as residuals and by-products of the polymerization procedure can accumulate in the pharmaceutical products through a process that follows a mechanism characterized mainly by solvation and diffusion processes. The transfer of these compounds may be direct (requiring contact) or indirect and they may further interact with the product, possibly forming unique degradation products [1,2]. Product packaging interaction studies address the contamination of the product by compounds derived from materials that come into contact with the product either during long-term storage or use [3].

Pharmaceutical analysis already entails trace level analytical applications for contaminants i.e., cross-contamination between products in the same production line [4,5]; however, migration studies differ in that the manufacturer may not be previously alert to its presence and, quite importantly, its removal may not be possible using cleaning/purging procedures. Migration studies involve the evaluation of the exchange between species taking place between a pharmaceutical product and the materials it is in contact with. Such materials are the production line materials employed in its manufacture, as well as the materials making up the final product packaging. Leachable species profiling studies focus on investigating contaminants in the actual product formulation. Extractable studies, on the other hand, are aimed at identifying the freely available substances present in a given material. In both cases, it is clear that the studies’ needs are both qualitative and quantitative [1,2]. As such, these studies employ screening methodologies based on analytical techniques that can give information on structure and quantify the species detected. Moreover, the techniques need to enable identification at low ppm levels—a requirement resulting from the safety concern thresholds suggested for potentially highly toxic impurities (genotoxic) [6]. Mass spectrometry coupled to liquid chromatography (LC-MS) meets the above requirements, at least for species belonging to the non-volatile category.

Studying the nature of materials enables the generation of a list of potential contaminants present. Considering the large diversity of extractable and leachable species observed, as well as the lack of commercially available reference standards, the listed compounds may serve as surrogate standards for performing semi-quantitation [7]. A common approach to quantitation is the use of internal standards (IS) [8]. Calibration and quantitation based on such substances, while allowing to cover for recovery losses and instrument response variability, still bear the risk of a higher margin of error since they are based on the presumption that the analytes have the same response factors as the IS and thus correspond to a semi-quantitation practice. This error in quantitation is not unacceptable, as long as it does not risk patient safety. More specifically, the “non-acceptable” part of this risk in quantitation is estimating the concentration of a species considerably below its true value, thus making it exempt from further evaluation, when in truth, it could be a risk.

For the above purpose, an uncertainty factor (UF) is used to divide the analytical evaluation threshold, thus lowering its value. This translates to accepting the risk of evaluating more species than necessary so as not to disregard something potentially dangerous [9,10]. The biggest contributor to analytical uncertainty in quantitation, as far as LC-MS-based methodologies are considered, is a difference in the propensity for the induction of a response. There are a few basic concepts that enable understanding of the sources of this difference. Mass spectrometry detection is dependent on the formation of ions. 

These ions are resolved (based on instrument capacity) based on their *m*/*z* values, while quantitation occurs based on their abundance (in terms of counts) at the detector. If all substances are capable of ionizing in the same way and producing the same number of ions in relation to their concentrations, then analytical responses would be independent of the substance and no quantitation error would occur. However, for mass spectrometric techniques, the response is indeed dependent on the respective species. Species arriving at the instrument’s ion source produce a number of ions that are associated with (a) their ability for ionization or adduct formation and (b) their lipophilicity (in a way that is indirect). Other factors that may also have a strong effect on the ionization efficiency of species include mobile phase composition, sample matrix, and ionization source design [11].

As far as (a) is concerned, this ability may range from null to high. Species with no polarizable center, i.e., hydrocarbons, have no ability to ionize or sustain the formation of a temporary ion (adduction) through electron dislocation/sharing. On the other hand, species with highly nucleophilic or electrophilic groups, e.g., amines and acids, may not require help to form ions. Species like alcohols, thiols, amides, esters, ketones, and aldehydes fall somewhere in between, based on the polarizability of the produced dipole.

Factor (b) is somewhat more complex to predict, and its impact on numerous aspects of the ionization process is considerable. These aspects include partitioning of charged droplets and dielectric constant of the eluting composition.

While several approaches for determining the uncertainty factor have been proposed, the most accurate proposition involves establishing Relative Response Factor (RRF) values for the different substances covered [12]. Relative response factors (RRFs) can be calculated using the following equation:

$$\mathrm{RRF}=\frac{{\mathrm{Slope}}_{\mathrm{Analyte}}}{{\mathrm{Slope}}_{\mathrm{Suggested}\;\mathrm{Internal}\;\mathrm{Standard}}}$$

where the slope of the analyte or internal standard signal versus concentration is estimated, through linear regression, in the linear range of the signal concentration plot.

The primary objectives of the current study were (a) establishment of one or more internal standards that will be subsequently introduced in routine analysis to improve the accuracy of semi-quantitative results and (b) establishment of a value for the uncertainty factor based on the use of those internal standards that provides sufficient coverage (i.e., enables flagging of the vast majority of compounds whose true concentrations are above the AET).

For the above purpose, the setup was designed to be highly similar to that used in routine analysis. The analysis was conducted under moderate changes to ensure that small variations in parameters, e.g., buffer concentration and %acetonitrile, do not result in great differences in response—or if they do, they could be considered during UF establishment. The exercise described in the reference protocol was also extended to GC-MS data. The data presented and used here-in were acquired under a different protocol for the performance of a test associated with the efficiency of the non-target screening algorithm. Nevertheless, since for the purposes of the aforementioned protocol the GC-MS and Head Space (HS) GC-MS methods analyzed solutions with variable concentrations allowing for the generation of a calibration curve, the data necessary for response factor generation were attained.

## 2. Materials and Methods

### 2.1. Representative Analytes

The substances included in the experimental design for the generation of the response factor database for LC-MS and GC-MS were purchased from Sigma-Aldrich (Darmstadt, Germany), Acros Organics (Geel, Belgium), Tokyo Chemical Industry Co., Ltd. (Haven, Belgium), and Toronto Research Chemicals (Toronto, ON, Canada). Due to the nature of the materials, purity varied (≥80%) and was taken into consideration during calculations pertaining to the concentration of analytes per level.

A set of 57 organic compounds, representing a diverse range of chemical functionalities and physical properties, were analyzed using LC-MS and used to create a response factor database [6]. The studied items are listed in Tables S1–S6 in the Supplementary Materials. These compounds were selected to represent a diverse range of polarity, chemical functionality, and ionization capacity on ESI. The majority are commonly encountered as extractables from common materials used to manufacture pharmaceutical packaging and in manufacturing and delivery systems [2]. The logP (logarithmic expression of the K_o/w_ octanol–water partition coefficient), polar surface, polarizability, and pK_a_ properties of the compounds are presented for information purposes and to confirm that a wide lipophilicity range is covered. To facilitate the preparation of standards for all these analytes, they were grouped into sets (Sets 1–5), as presented in Table S7 in the Supplementary Materials.

A set of 43 organic compounds, representing diverse chemicals in terms of characteristic moieties but more importantly in terms of boiling points (as a parameter associated with the volatility of a substance) and logP (as a parameter of lipophilicity), were analyzed using GC-MS and used to create a response factor database similar to that created using the LC-MS-based process. The studied items are listed in Table S8 in the Supplementary Materials along with data on the aforementioned physicochemical parameters, their allocated set (they were split into 3 sets) and the *m*/*z* values of the fragment ions of higher relative abundance from their fragmentation spectrum.

### 2.2. Reagents

Reagents such as ammonium formate and solvents such as water, methanol, and LC-MS grade acetonitrile were purchased from Sigma-Aldrich (Darmstadt, Germany). Ethyl acetate (analytical reagent grade) was obtained from Fisher Chemicals (Bremen, Germany). Propan-2-ol and *n*-pentane for trace analysis were purchased from Sigma-Aldrich (Darmstadt, Germany).

### 2.3. Optimization of the MS Signal

When calculating an analyte’s response, one should take into consideration dependencies on components of the analytical setup that are expected to vary to some extent. Buffer concentration and % content in a co-solvent are such potential parameters for which it is necessary to evaluate whether they affect the response (MS signal) significantly [13]. Buffer concentration may affect the response of ammonium-adduct-forming compounds or compounds able to form both adducts and pseudomolecular ions due to the response being split into more than one ion. Furthermore, higher %acetonitrile fractions may be necessary to allow elution of species of considerable lipophilicity; however, regardless of this chromatographic effect, the reagent may affect ionization efficiency due to the sharp decrease in the medium’s surface tension, as well as an increase in its evaporation rate.

To investigate the effects of those two parameters on analyte response, a 2^2^ full factorial design was drafted to estimate both the main effects and the interactions between factors [14]. In these designs, each factor has an upper and lower level, and a center point can be introduced. The levels of both factors used in the current study are presented in Table 1. 

The data acquired were ultimately handled using response surface methodology (RSM). A mathematical model that allows for processes such as value maximization, minimization, and specific target value input was used [15]. Visualization of the created response surface also has added value because it makes it easy, upon placing value constrains for the response targets, to see the “space/range of variable values” within which a desired outcome is attained.

In this case, the desired outcome is proper classification of a target as “above AET” when this is the actual scenario and is not a matter of limiting response variation per se. Practically, the response may vary as long as it does so in a manner that is homoscedastic to the internal standard used in the evaluation. Heteroscedastic behavior is the actual source of erroneous classification, meaning the increase in internal standard response simultaneously with a decrease in analyte response.

### 2.4. LC-MS Method

A Thermo Scientific™ Accela ultra-performance liquid chromatographic system (San Jose, CA, USA) composed of a pump, an autosampler, and a PDA 80 Hz detector coupled to an LTQ-Orbitrap mass spectrometer employing a Heated Electrospray source was utilized. Chromatographic runs were performed using a Thermo Scientific Acclaim™ USP L7 (C8) phase column (50 mm × 2.1 mm, 5 μm particle size) maintained at 40 °C. Total run time lasted 23 min and the mobile phase consisted of 5 mM ammonium formate aqueous solution (Mobile Phase A) and acetonitrile/methanol 10/90% *v*/*v* (Mobile phase B). The gradient program is presented in Table 2. The injection volume was 5 μL and the autosampler temperature was set at 25 °C. 

The optimal values/settings for the MS parameters were: positive/negative polarity, full scan for acquisition mode (scan range 50–1200 *m*/*z*) and targeted MS^2^ as fragmentation pattern data acquisition mode (as a secondary experiment performed independently), 45 and 25 arbitrary units for sheath and auxiliary gas pressure, respectively, capillary temperature of 320 °C, source heater temperature of 250 °C, and S-Lens RF Level of 50.0%. 

### 2.5. GC-MS

A Shimadzu gas chromatography system (Duisburg, Germany) consisting of a gas chromatograph and an autosampler with headspace and liquid injection capabilities coupled to a mass spectrometer employing an electron ionization (EI) source was utilized. Chromatographic separations were performed using an Agilent™ HP-5ms UI (5% Phenyl)-methylpolysiloxane phase, 30 m × 0.25 mm, 0.25 μm thickness (G27 USP category) column obtained from Pegasus S.A. (Athens, Greece). Helium, at a flow rate of 1.60 mL/min, was used as a carrier gas. The temperature at the injection port was set to 305 °C and the injection mode was splitless, with a sampling time of 1.05 min. The column temperature program is given in Table 3.

The following MS parameter settings were used: 315 °C for interface temperature, 250 °C for ion source temperature, 35–800 *m*/*z* for Scan range, and a run time of 3.0–31.5 min. 

### 2.6. Preparation of Standard Solutions for LC-MS Analysis

Standard solutions for the different substances were prepared from intermediate solutions with a concentration of 10 μg/mL diluted in methanol. The intermediates were prepared from stock solutions of each individual substance, weighed (10 mg), and accurately diluted to a final volume of 5.0 mL using ethyl acetate (for a concentration of 2000 μg/mL. The standard solutions prepared from the intermediate solutions of each “set” (as described in Table 1) had concentrations of 0.40 μg/mL (Standard A), 0.75 μg/mL (Standard B), 1.00 μg/mL (Standard C), 1.50 μg/mL (Standard D), and 2.00 μg/mL (Standard E). The solution’s final composition was 20/80 methanol/water (*v*/*v*).

### 2.7. Preparation of Standard Solutions for GC-MS Analysis

The standard solutions for the different substances were prepared from intermediate solutions with a concentration of 10 μg/mL diluted in propan-2-ol. The intermediates were prepared from stock solutions of each individual substance, weighed (10 mg), and accurately diluted to a final volume of 5.0 mL using ethyl acetate to get a concentration of 2000 μg/mL. The standard solutions prepared from the intermediate solutions of each “set” (as described in the tables above) had concentrations of 0.40 μg/mL (Standard A), 0.75 μg/mL (Standard B), 1.00 μg/mL (Standard C), 1.50 μg/mL (Standard D), and 2.00 μg/mL (Standard E). The diluent in these preparations was 50/50% propan-2-ol/water (*v*/*v*). The standards were extracted in duplicate in a 2:1 ratio with *n*-pentane; 4 mL of the propan-2-ol/water mixture standard was extracted with 2 mL of pentane two successive times. A biphasic system is formed between pentane and the propan-2-ol/water mixture, with *n*-pentane in the upper phase. For each concentration level, the *n*-pentane phase was quantitatively acquired in triplicate, upon instrument mass calibration and system precision testing, to confirm system readiness.

## 3. Results and Discussion

### 3.1. LC-MS Analysis

The LC-MS method corresponds, as expected, to a gradient method that increases from a low organic percentage of 2% to a final of 95%. Wide-scope screening methods are inevitably associated with gradient methods since the lipophilicity of target analytes differs (or is expected to). Moreover, they usually correspond to relatively low-slope gradients since the resolving power of the chromatographic separation method—otherwise referred to as capacity for chromatographic separations—is roughly estimated as described in [16]:

$$\mathrm{Capacity}=\frac{\mathrm{Total}\;\mathrm{Chromatographic}\;\mathrm{Time}}{\mathrm{Average}\;\mathrm{Peak}\;\mathrm{Width}\;\mathrm{at}\;\mathrm{Medium}\;\mathrm{height}}=\frac{23.0\;\min}{0.13\;\min}\approx 177\;\mathrm{peaks}\;\mathrm{per}\;\mathrm{chromatogram}$$

The value has little practical importance for the simple reason that it is impossible to actually predict whether the unknown analytes included in samples belong to variant groups and will thus provide a “dispersed” profile across the total chromatographic time or they are “dense” in highly similar species that will amass in a specific region. Methods through which detection is achieved through mass spectrometers do not depend strongly on chromatographic separation. This is a direct consequence of the high specificity achievable by mass spectrometers (even more so by high resolution MS systems) that allow the extraction of information for a single ion from the total chromatogram containing millions of recorded ions.

To make a simplistic reference to the process through which this is achieved, the following can be described [17,18]: The analytes are roughly separated chromatographically and arrive at the instrument’s ion source probe (in this application heated electrospray). They are incorporated into droplets bearing a number of ions (cations or anions depending on the mode of operation/polarity of the voltage applied). The ions are formed under the effects of a voltage applied between the probe and the ion transfer tube—some species are directly ionizable, while others enter an ionized state through adduct formation with mobile phase ions (the simplest being H^+^ for positive ionization).

The ions are centered through lenses and scanned through the application of electromagnetic force fields. Briefly, the system can discriminate between different ions and record their individual intensities based on their behaviors within the fields. The system employed in this application corresponds to an orbitrap. The orbitrap achieves this discrimination through orbital frequency—the mass to charge (*m*/*z*) value of an ion determines its frequency of orbital movement within the electromagnetic field, while the number of ions with the same frequency is determined through inductive current intensity measurement (a moving ion is a current) [19]. As a result, the total ion chromatogram acquired from a mass spectrometer shows the sum of intensities of ions trapped in orbits, yet by observing the spectrum at a given time, one can annotate the ions that compose it and extract the data for the one desired.

To place it in context, even if multiple substances are eluted at a given time, it is possible for the user of a mass spectrometer to “extract” the chromatogram of the peak for one of them. The only reason to setup a chromatographic method with high capacity is the antagonistic behavior of analytes during ionization. A method achieving a fair chromatographic separation enables reduction of the occurrence of co-elutions that make ion abundance a limitation for response.

**Figure 1:**
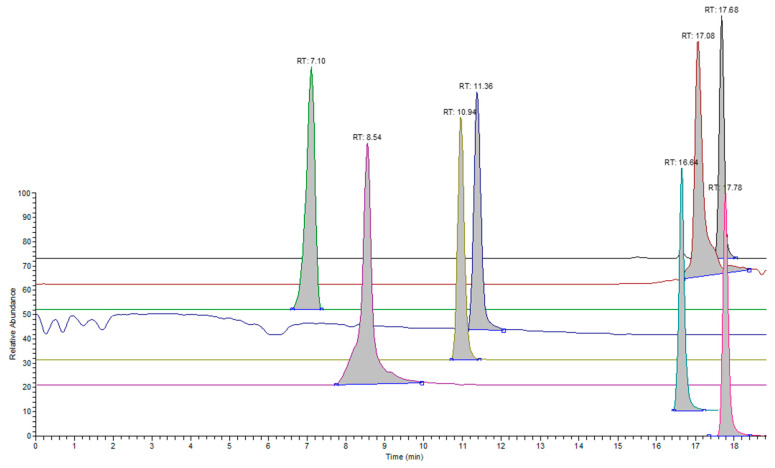
Extracted ion chromatograms for representative analytes in set 1 under the positive ionization mode. The following analytes are presented from back to front: bis(2-ethylhexyl) hexanedioate, CAS: 103-23-1 (black), (Z)-octadec-9-enamide, CAS: 301-02-0 (dark red), 2-methylbenzenesulfonamide, CAS: 88-19-7 (green), N-butylbenzenesulfonamide, CAS: 3622-84-2 (blue), Benzenesulfonylbenzene, CAS: 127-63-9 (mustard yellow), 1,2-bis(2-methylphenyl) guanidine, CAS: 97-39-2 (violet), dibutyl decanedioate, CAS: 109-43-3 (teal), and tris(4-tert-butylphenyl) phosphate, CAS: 78-33-1 (pink).

**Figure 2:**
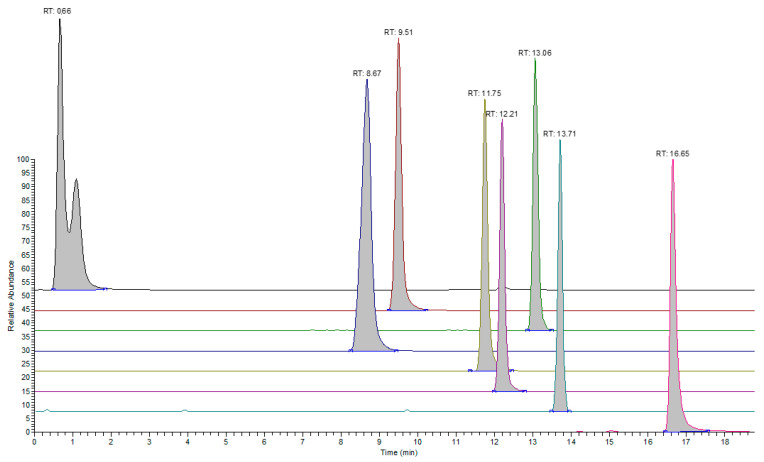
Extracted ion chromatograms for representative analytes in set 2 under the positive ionization mode. The following analytes are presented from back to front: 2,2,6,6-tetramethylpiperidin-4-ol, CAS: 2403-88-5 (black), Triethyl phosphate, CAS: 78-40-0 (dark red), Methyl benzenesulfonate, CAS: 80-18-2 (green), 4-(4-hydroxyphenyl) sulfanylphenol, CAS: 80-09-1 (blue), N,N-diethyl-3-methylbenzamide, CAS: 134-62-3 (mustard yellow), [2-(hydroxymethyl)-3-prop-2-enoyloxy-2-(prop-2-enoyloxymethyl)propyl] prop-2-enoate, CAS: 4986-89-4 (violet), bis(3,5-dimethylphenyl) phosphane, CAS: 71360-06-0 (teal), and octadecan-1-amine, CAS: 124-30-1 (pink).

**Figure 3:**
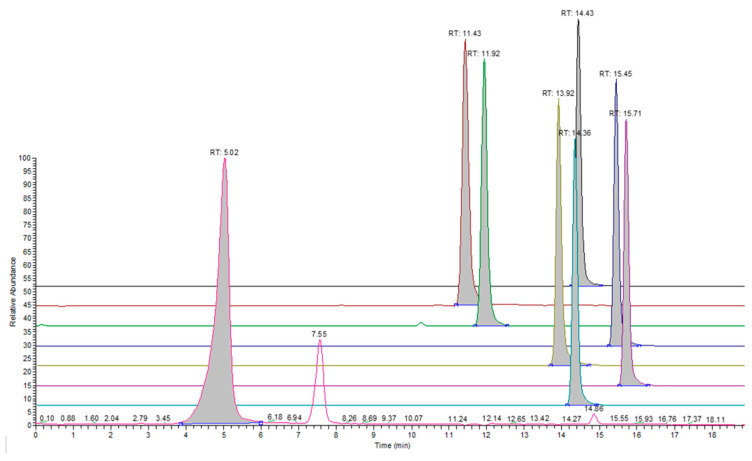
Extracted ion chromatograms for representative analytes in set 3 under the positive ionization mode. The following analytes are presented from back to front: 3,5-Di-tert-butyl-4-hydroxybenzaldehyde, CAS: 1620-98-0 (black), Octanamide, CAS: 629-01-6 (dark red), 2-[2-(2-methylprop-2-enoyloxy) ethoxy] ethyl 2-methylprop-2-enoate, CAS: 2358-84-1 (green), Tris(2-butoxyethyl) phosphate, CAS: 78-51-3 (blue), 2-[dodecyl(2-hydroxyethyl) amino] ethanol, CAS: 1541-67-9 (mustard yellow), tris(2-methylphenyl) phosphate, CAS 78-30-8 (violet), Triphenyl phosphate, CAS: 115-86-6 (teal), and oxepan-2-one, CAS: 502-44-3 (pink).

Table 4 contains the responses per ppm of analyte concentration for the analytes detected under the positive ionization mode (sets 1–3). System suitability testing was carried out prior to the analysis of samples and involved employing the 0.40 μg/mL test solution (Standard A) for sets 1 and 4. The %RSD values for the response of analytes were ≤10%. Characteristic overlaid chromatograms are presented for analytes in different sets (Figure 1, Figure 2 and Figure 3).

The population included was very diverse, as reflected in the statistical descriptive parameters of the population. The %RSD for the response slope values is equal to 136.1%, with a median of 10,122,291.2 peak area units per ppm concentration and a mean/average of 33,849,251.7 peak area units per ppm concentration.

**Figure 4:**
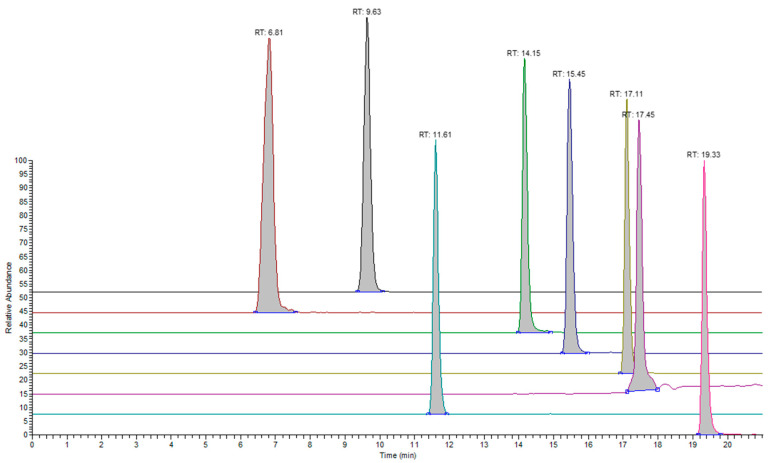
Extracted ion chromatograms for representative analytes in set 4 under the negative ionization mode. The following analytes are presented from back to front: Bis(4-methoxyphenyl) phosphinic acid, CAS: 20434-05-3 (black), 2,4-Dichlorobenzoic acid, CAS: 50-84-0 (dark red), 2,6-ditert-butyl-4-(hydroxymethyl) phenol, CAS: 88-26-6 (green), 16-Hydroxyhexadecanoic acid, CAS: 506-13-8 (blue), (1R,4aR,4bR,10aR)-1,4a-dimethyl-7-propan-2-yl-2,3,4,4b,5,6,10,10a-octahydrophenanthrene-1-carboxylic acid, CAS: 514-10-3 (mustard yellow), hexadecanoic acid, CAS: 57-10-3 (violet), 2,6-dibromo-4-[3-(3,5-dibromo-4-hydroxyphenyl)-1,1-dioxo-2,1lambda6-benzoxathiol-3-yl] phenol, CAS: 115-39-9 (teal), and [3-[3-(3,5-ditert-butyl-4-hydroxyphenyl) propanoyloxy]-2,2-bis[3-(3,5-ditert-butyl-4-hydroxyphenyl) propanoyloxymethyl] propyl] 3-(3,5-ditert-butyl-4-hydroxyphenyl) propanoate, CAS: 6683-19-8 (pink).

**Figure 5:**
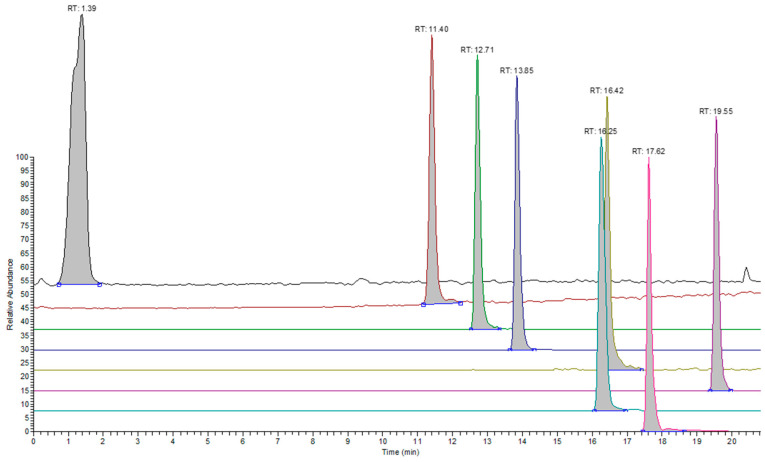
Extracted ion chromatograms for representative analytes in set 5 under the negative ionization mode. The following analytes are presented from back to front: 2-hydroxy-2-phenylacetic acid, CAS: 90-64-2 (black), 2-Ethylhexanoic acid, CAS: 149-57-5 (dark red), 2-Phenylphenol, CAS: 90-43-7 (green), 2-(2-ethylhexoxycarbonyl) benzoic acid, CAS: 4376-20-9 (blue), 2,6-ditert-butyl-4-methylphenol, CAS: 128-37-0 (mustard yellow), 4-[[3,5-bis[(3,5-ditert-butyl-4-hydroxyphenyl) methyl]-2,4,6-trimethylphenyl] methyl]-2,6-ditert-butylphenol, CAS: 1709-70-2 (violet), 2-[2-[2-[3-(3-tert-butyl-4-hydroxy-5-methylphenyl) propanoyloxy] ethoxy] ethoxy] ethyl 3-(3-tert-butyl-4-hydroxy-5-methylphenyl) propanoate, CAS: 36443-68-2 (teal), and 2-tert-butyl-6-[(3-tert-butyl-5-ethyl-2-hydroxyphenyl) methyl]-4-ethylphenol, CAS: 88-24-4 (pink).

Similarly, data for the negative ionization mode (sets 4 and 5) are presented in Table 5 and relevant chromatograms are presented in Figure 4 and Figure 5. The same observation is applicable to the species observed under the negative ionization mode. The %RSD for the response slope values is equal to 138.3%, with a median of 2,178,866.1 peak area units per ppm concentration and a mean/average of 2,814,174.3 peak area units per ppm concentration.

### 3.2. GC-MS Analysis

The extraction procedure described in Section 2.7 means that the standards acquired and analyzed for response propensity determination of the substances included incorporates the extraction recovery factor [20]. This was an informed choice, which arises from practice. Solvents most commonly used in extractable species profiling include alcohol–water mixtures and water-based buffers. Rarely is a very lipophilic solvent (e.g., hexane or dichloromethane) a suitable medium with relevance to the extraction propensity of a pharmaceutical product [21].

In leachable species profiling, the pharmaceutical product is handled as a matrix from which the target analytes need to be acquired. Given the relevance pharmaceuticals need to retain with biological environments and matrices, most of them are water-based systems. The presence of surfactants, proteins, and alcohol co-solvents increases the propensity for extraction of pharmaceuticals beyond that of “pure water-based buffers”. This is also the reason why the standards were prepared in a 50/50 *v*/*v*% propan-2-ol/water. The potential “antagonism” of the medium against pentane to retain the analytes was considered at its worst-case.

Table 6 includes the response per ppm of analyte concentration for the analytes detected in the different sets (1–3) used in GC-MS. System suitability testing was carried out prior to the analysis of samples and employed the 0.40 μg/mL test solution (Standard A) for set 1. The %RSD value for the response of analytes was ≤11%.

### 3.3. Descriptive Statistics for LC-MS Data

**Figure 6:**
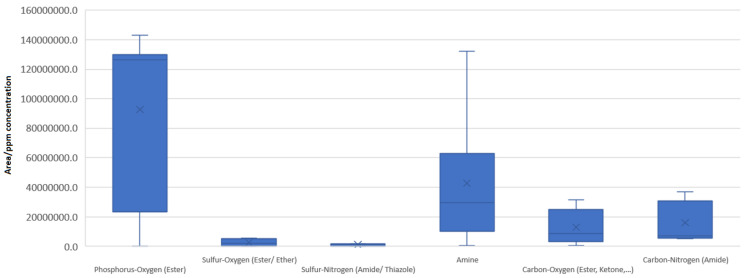
Box plot presentation of the descriptive statistical parameters of the chemical categories included for evaluation under the positive ionization mode. The upper and lower bases of the rectangle (box) are set at the 1st and 3rd quartile. The line within the box represents the median. Lines extend outside of the box to mark outlier values of the base population.

The first step in the evaluation is based on descriptive statistics data and strategy generation. If the substances are split into their chemical classes, the box plots presented in the following figures can be created. By placing box plots of different chemical categories next to one another (Figure 6), it is possible to make comparisons i.e., populations presenting a similar span range or different range but a similar median. Commencing with the positive mode of ionization, the bullets below summarize some of the observations made based on the plots of the groups:

1. The substances that are based on polarizable carbon–oxygen or carbon–nitrogen bonds (i.e., carbamides, esters, ketones) are highly relevant in terms of response. Both have small variability and their medians are approximately equal, implying that the polarizability of the bond is similar for both pairs: carbon–oxygen and carbon–nitrogen.
2. The substances corresponding to a polarizable phosphorus–oxygen bond appear to have one of the highest variabilities. This is due to the unstable bis(2-ethylhexoxy)-oxophosphanium species included. The median of the population is over 3-fold higher than for all other groups.
3. Amines, corresponding to ionizable nitrogen-based substances, have a median that is higher than that of carbonyl and carbamide species. They, however, exhibit a high variability that is not due solely to outlying values but rather a natural diversity (the box is wider, not just the extended lines).

4. All sulfur-based polarizable bonds (sulfate esters, sulfur ethers, sulfonamides, etc.) have low but similar responses compared with other chemical categories, meaning that sulfur–oxygen and/or sulfur–nitrogen bonds do not differ much in propensity.

**Figure 7:**
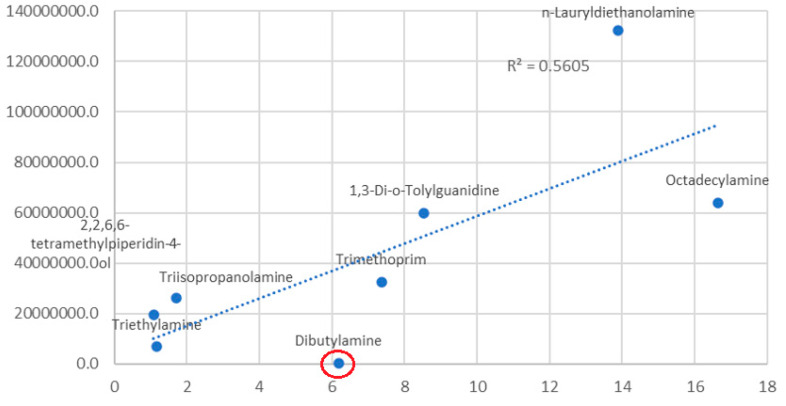
Linear correlation plot of retention time (*x*-axis) versus the response slope of the analyte (*y*-axis).

Focusing on the amine chemical group, different graphic presentations enable evaluation of the source of the high variation observed. Correlation between retention time and response supports the hypothesis that the elution environment affects the response. Dibutylamine (*N*-butylbutan-1-amine, CAS: 111-92-2) has an outlier value to the rough correlation observed (Figure 7). The above explanation does not fall far outside the phenomena that have been described for electrospray ionization, i.e., the lower efficiency of desolvation and ion/adduct transition to the gas phase for polar substances.

**Figure 8:**
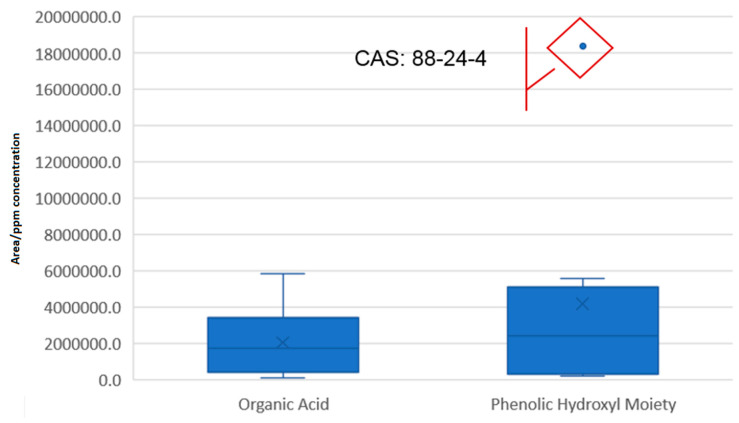
Box plot presentation of the descriptive statistical parameters of the chemical categories included for evaluation under the negative ionization mode.

Following up with the groups from the negative ionization mode, it is evident that there is an outlier value belonging to 2-tert-butyl-6-[(3-tert-butyl-5-ethyl-2-hydroxyphenyl) methyl]-4-ethylphenol, CAS: 88-24-4. When rejected, the %RSD value for the remaining species falls to 86.2%, while the span (max/mix ratio) is reduced to a factor of 10^2^ from a previous value of 3 × 10^2^. The two groups have a similar median, although the organic acid population has a lower response variability (Figure 8). The Q_1_ (1st quartile) value is also fairly similar. Due to their similarities, it is logical to assume that a single substance can effectively help in the quantitative estimation of either categories.

### 3.4. Strategy for LC-MS Analysis

Amines are the primary chemical category analyzed using LC-MS under the positive mode of ionization. They are notorious of their ability to degrade semi-polar and non-polar sorbents of GC columns through irreversible binding and, like many substances bearing moieties amenable to hydrogen or ion bonding, they require high energy for gas transition (relative to other substances of similar MW). The same applies to organic acids detected under the negative ionization mode of LC-MS.

An additional chemical category that should be covered is organic species with carbon–oxygen or carbon–nitrogen polarizable bonds [22]. This group of substances contains the majority of potential analytes in migration studies. The reason is quite logical if one considers where the contaminants come from: polymerization processes.

Some of the functional polymers currently applied in the industry, including nylon, polyesters, and olefin blends based on poly(ethylene) or poly(propylene) mixtures, are based on esterification and amidification procedures. In addition, many other chemistries employ ketone, phenol or benzoyl peroxides for polymerization initiation. Smaller MW esters/ketones are used as dispersion solvents during the process; acids, amines, and alcohols are used for polymerization control and cross-linking; amides are used as slip agents. Functional additivation is also commonly used to improve the resistance of the final product to degradation based on its expected exposure to environmental conditions or mechanical stress.

While the above categories (organic substances with a polarizable carbon–oxygen or carbon–nitrogen bond) are partially amenable to detection using GC-MS, redundancy is desirable in screening methodologies because it acts as a safety measure to address cases with severe detectability issues caused by a product matrix.

The performance of species with a phosphorus–oxygen polarizable bond (higher response slopes) places them in a favorable position in terms of detectability. Should one cover for the other chemical classes, the phosphorous polarizable bond category will always be overestimated and thus reported for further evaluation even when below the analytical evaluation threshold. On average a phosphorus-polarizable bond-bearing substance can elicit a response equivalent to amines or polarizable-carbon-bond-bearing chemicals, even at a concentration that is five times lower. While this increased risk of “false positive” results is generally acceptable as a “producer’s risk” rather than a “consumer’s risk”, it can be averted by the inclusion of a suitable representative for quantitation of such substances upon their recognition.

**Figure 9:**
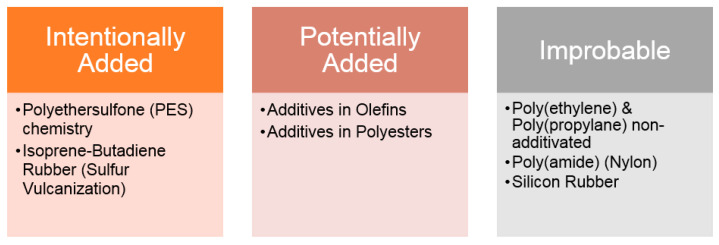
Classification of materials.

Sulfur-based polarizable bonds, on the other hand, have a lower propensity for response and thus, a higher risk of avoiding detection. The risk is partially mitigated by their amenability to detection using GC-MS. Nevertheless, as previously mentioned, redundancy is a desired characteristic, and this applies to this category as well due to its bad toxicological profile. Sulfur-based chemicals account for multiple sensitization events. The sensitization threshold is 3-fold higher than the genotoxicity threshold typically applied (Safety Concern Threshold (SCT) of 5 μg/day instead of 1.5 μg/day). Depending on the strategy on the margin of safety provided by the “difference” between the two SCTs would be hazardous because the SCT used in the AET calculation can change for certain pharmaceuticals, e.g., sub-chronic administration and drugs with a weekly or monthly dose regimen. Instead, it is recommended to consider the availability of such substances in different materials. Sulfur-based substances arise only through additivation. As a result, it is possible to classify materials per their propensity to contain such species in the manner presented in Figure 9.

It is recommended that a representative of the polarizable sulfur–oxygen group of chemicals is used in experiments designed to address the profiles of materials in the potentially or intentionally added categories. The analyte selected should be considered for the initial evaluation step of the process instead of the substance typically used as an internal standard—at least for compounds that fit into the isotopic profile of sulfurous substances. Considering all of the above, the criteria for internal standard selection in both positive and negative ionization modes are:

- The substance should present a response approximately equal to the Q_1_ (1st quartile) of the critical compound population to be covered. The positive ionization includes amines and species with polarizable carbon–oxygen and carbon–nitrogen bonds (amides, esters, ketones, etc.), while the negative ionization includes both acids and phenolics.
- The substance should, ideally, not belong to a typically expected analyte.

The analyte selected for use as an internal standard in positive ionization is pentaerythritol tetraacrylate (IUPAC: [2-(hydroxymethyl)-3-prop-2-enoyloxy-2-(prop-2-enoyloxymethyl) propyl] prop-2-enoate, CAS: 4986-89-4). The substance has a response slope of 6,454,678.4 peak area per ppm of concentration; the population’s Q_1_ value is 6,253,579.5 (+3.2% difference).

The analyte selected for use as an internal standard in negative ionization is bromophenol blue (IUPAC: 2,6-dibromo-4-[3-(3,5-dibromo-4-hydroxyphenyl)-1,1-dioxo-2,1 lambda 6-benzoxathiol-3-yl] phenol). The substance has a response slope of 585,533.2 peak area per ppm of concentration; the population’s Q_1_ value is 376,976.9 (+55.7% difference).

### 3.5. Evaluation for LC-MS Analysis

**Figure 10:**
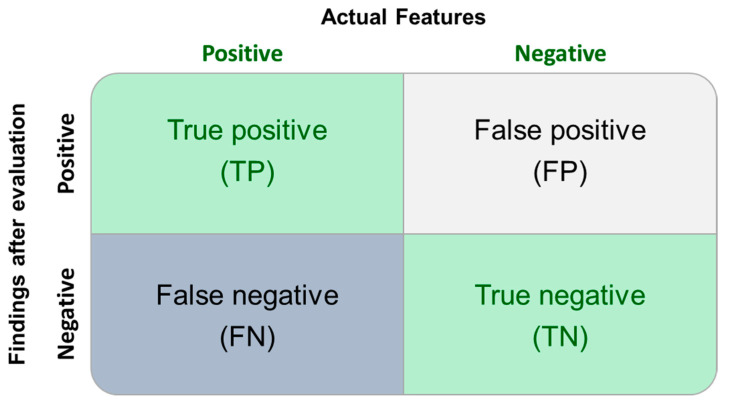
Diagram showing the classification of species: the adjectives true and false characterize the results of the classification against “the fact”, while the nouns positive and negative characterize the annotation provided by the process as per the characteristic used (>AET is positive; <AET is negative).

To evaluate the impact of the above selection in the classification of substances, the substances were used for response factor (relative response) generation. When the acquired value is ≥1.0, the substance is reported for further evaluation. If the substance has a value < 1 but ≥0.5, it would be reported for further evaluation under the typical design that incorporates a 0.5 uncertainty factor in the calculation of the AET. If the substance’s relative response is <0.5, the incorporation of the typical 0.5 uncertainty factor is not successful at protecting against the “mis-classification” of the substance (Figure 10).

Sensitivity, defined as the number of positives as per the evaluation process that are in fact positive, proceeds based on the data available and can be calculated using the equation:

$$\mathrm{Sensitivity}=\frac{\mathrm{TP}}{\mathrm{TP}+\mathrm{FN}}$$

Table 7 and Table 8 indicate the relevant calculations of both LC-MS operation modes.

### 3.6. Descriptive Statistics of GC-MS Data

**Figure 11:**
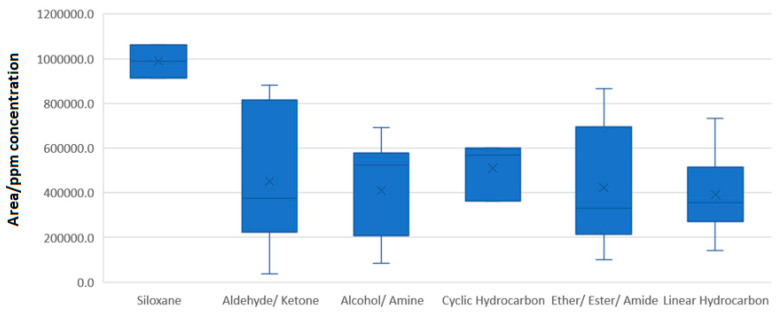
Box plot presentation of the descriptive statistical parameters of the chemical categories included for evaluation using GC-MS.

The data acquired for species using GC-MS suggest that the overall population of substances has an average response of 430,250.9 area units per ppm of concentration, with a %RSD of 41.3%. It is evident that the overall variability is much lower compared with that observed using LC-MS. Splitting the population into sub-groups associated with their chemical categories (characteristic chemical moiety present) shows that siloxanes have a higher propensity for responses, while the remaining carbon-based species have a similar range, meaning that it becomes simpler to designate a substance suitable for their evaluation (Figure 11).

### 3.7. Strategy for GC-MS Analysis

The basic population of species for which detection needs to be assured is hydrocarbons. The reason behind this is that the different methods employed are complementary in their applicability domains. Hydrocarbons—linear or cyclic, saturated or unsaturated—bear no polarizable bonds and are thus capable of escaping detection under LC-MS analysis. GC-MS is, thus, often used for their detection. Highly volatile ketones, aldehydes, alcohols, and ethers are secondary groups of species that should be analyzed using GC-MS. The last group is siloxanes. Given the higher relative propensity for siloxane responses, it is logical that they need not guide the process of selection for the internal standard.

Similar to the LC-MS process, the analyte selected for use as an internal standard should be close to the population’s Q_1_ value—in this case, 321,790.9 area units per ppm of concentration. The analytes dodec-1-ene (CAS: 112-41-4) and tetradec-1-ene (1120-36-1) are very close to the desired value; however, being hydrocarbons (specifically alkenes), specificity issues arising in extracts are expected. As a result, the species 1-fluoro-2-phenylbenzene (CAS: 321-60-8) was also selected for evaluation as an alternative.

### 3.8. Evaluation for GC-MS Analysis

Similar to LC-MS analysis, Table 9 shows the relevant calculations using GC-MS.

It is evident that the performance of 1-fluoro-2-phenylbenzene (CAS: 321-60-8) is sub-optimal compared with that of dodec-1-ene (CAS: 112-41-4); however, should specificity issues arise, its use may be necessary even if reconsideration of the UF value, down to 0.40 instead of 0.50, is required.

### 3.9. Robustness Testing of the LC-MS Method

As previously mentioned, evaluation of the LC-MS methodology included robustness testing under a 2^2^ factorial design for the creation of a design space within which the user of the method can proceed with intended changes to the factors of mobile phase composition as per %acetonitrile and buffer concentration without affecting evaluation of the analytes.

The analytes included in the design for response factor generation were analyzed under the different setups and their responses were found to vary (as expected). Variation under positive ionization ranged from as low as 12.3% (%RSD between analyte response in the experiments) to as much as 119.0%, while in negative ionization, variation ranged from 10.5 to 116.6% (Tables S9 and S10, respectively, in the Supplementary Material).

First, the response factors of the different analytes were generated using the response value of the internal standard chosen. Species for which the variation in the factor generated in all experiments is ≥0.5 are considered “successful evaluation cases”. Technically, these substances co-vary with the internal standard within the entire space covered in the design.

Species for which the response factor generated is ≤0.5 under all variations correspond to “consistently misallocated cases”. The species that fall within the “equivocal zone” of the design space are those that enable identification of its bounds.

Under the positive ionization mode, the species falling under equivocal (having some correct and some wrong allocations) were: octanamide, CAS 629-01-6, (Z)-docos-13-enamide, CAS 112-84-5, oxepan-2-one CAS 502-44-3, 4-(4-hydroxyphenyl) sulfonylphenol CAS 80-09-1, and benzenesulfonylbenzene CAS 127-63-9.

The response surface methodology (RSM) provided a solution for simultaneous optimization of the response factor of the analytes—6.72 mM buffer concentration and 20% acetonitrile—in order to obtain a response factor ≥ 0.5 for all substances. The diagrams of factor importance in all cases showed that buffer concentration was of negligible importance (statistically on a 95% significance), while the acetonitrile %content of mobile phase B was critical.

**Figure 12:**
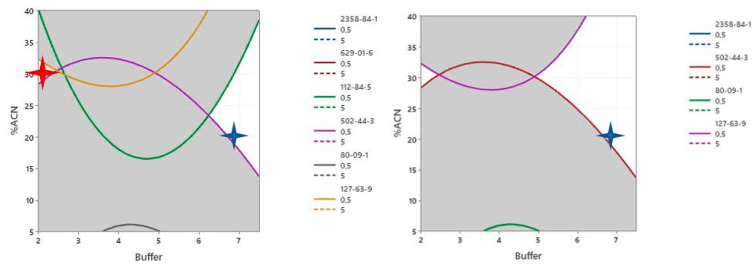
Contour plot of the design space for critical cases in positive ionization generated using the RMS methodology.

The contour plots in Figure 12 show two “pockets” within the design space (the white spaces). The blue star shows the solution that was provided by the model.

Under negative ionization mode, the species falling under equivocal (having some correct and some wrong allocations) were: 2-sulfanylbutanedioic acid, CAS 70-49-5, 4-hydroxy-3-methoxybenzoic acid, CAS 121-34-6, 2,4-dichlorobenzoic acid, CAS 50-84-0, terephthalic acid CAS 100-211-0, 2-ethylhexanoic acid CAS: 149-57-5, 2-phenylphenol CAS: 9043-7, and 2,6-ditert-butyl-4-methylphenol CAS 128-37-0.

The response surface methodology (RSM) [23] provided a solution for simultaneous optimization of the response factor of the analytes—3.72 mM buffer concentration and 25.8% acetonitrile in Mobile Phase B—in order to obtain a response factor ≥0.5 for all substances. Again, in all cases, the diagrams of factor importance showed that acetonitrile %content of mobile phase B was the critical factor.

**Figure 13:**
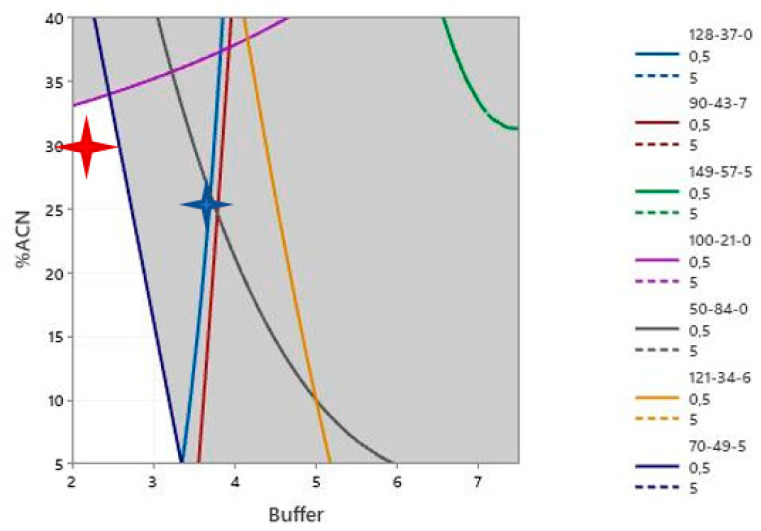
Contour plot of the design space generated for critical cases in negative ionization using the RMS methodology.

The contour plot (Figure 13) shows one “pocket” within the design space (the white space). The blue star shows the solution that was provided by the model. Desirability affects the solution provided by the model, which explains why the model may provide a solution that is not optimal for some of the substances (CAS: 128-37-0 in the case below).

As per the results of the design surface analysis, the center conditions could be moved from the 10% acetonitrile/5 mM buffer concentration combination to a 2.25 mM ammonium formate buffer concentration and a 30/70% *v*/*v* acetonitrile/methanol in mobile phase B (red star in the design spaces).

The solution provides good results in both positive and negative ionization and provides “a safe space” since the proper annotation is not affected within 2–2.5 mM buffer concentration and 27–32.5% *v*/*v* acetonitrile in mobile phase B. The space is too limited to enable modification but is sufficient for controlling against minor unexpected changes during preparation.

## 4. Conclusions

Within the presented work, a stratified sampling procedure was used to select representative analytes for analyte response evaluation. The stratification was based on physicochemical properties relevant to each methodology (logP, volatility, and ionizable and polarizable chemical moieties). The responses of the selected substances were evaluated using LC-MS and GC-MS. Based on the response slopes of the analytes, a strategy was formulated, internal standards for primary compound evaluation were selected, and the protective power of the process was estimated, taking into consideration the currently proposed uncertainty factor and/or proposing the establishment of a different value.

The internal standards selected for LC-MS allowed proper classification of other species as “above the AET” for over 76.9% of chemicals when taking the standard UF value of 0.50 (95.2% for critical populations in positive ionization, 86.4% for all populations in negative ionization) into consideration within the context of the instrumental parameters used for detection and chromatographic separation.

The internal standards selected for GC-MS were dodec-1-ene (CAS: 112-41-4) and 1-fluoro-2-phenylbenzene (CAS: 321-60-8), the latter being a second choice due to interference being a realistic scenario in the case of the hydrocarbon. Employing 1-fluoro-2-phenylbenzene requires reducing the UF to 0.40 to obtain results similar to those obtained using dodec-1-ene—achieving a process sensitivity in the 92% range.

Due to the high variability observed in LC-MS, the generation of a design space in which the evaluation is not compromised—intentionally or not—by changes in critical parameters of the chromatography and ionization was defined. Based on the application of a minimal 2^2^ factorial design and its evaluation using RMS, it was possible to find an optimal condition space with the required “ruggedness” to unintended but expected changes occurring.

Future endeavors will include evaluation of responses using (HS)GC-MS applications and generation of models for response and/or retention—structure correlation.

## Figures and Tables

**Table 1:** Experimental parameters and their levels under investigation.

Parameter	Lower Level (−1)	Central Level (0)	Upper Level (+1)
Ammonium Formate (mM)	2.0	5.0	7.5
%Acetonitrile in Mobile Phase B	5%	10%	40%

**Table 2:** Gradient program for LC-MS.

Time	%B	Flow
0.0	2	0.25
2.0	2	0.25
16.3	95	0.25
19.0	100	0.40
23.0	100	0.40

**Table 3:** Column temperature program.

Rate (°C∙min^−1^)	Temperature (°C)	Hold Time (min)
-	40	1.5
10.0	130	0.0
15.0	260	1.5
15.0	310	7.5

**Table 4:** Data pertaining to the chromatographic retention of species evaluated under positive ionization, along with response slope values from individual calibration curves.

CAS	Name	Retention Time (min)	Response Slope (Peak Area/ppm Concentration)
122-20-3	1-[bis(2-hydroxypropyl) amino] propan-2-ol	1.71	26,366,509.7
111-92-2	*N*-butylbutan-1-amine	6.19	406,919.2
88-19-7	2-methylbenzenesulfonamide	7.13	24,803.8
738-70-5	5-[(3,4,5-trimethoxyphenyl) methyl] pyrimidine-2,4-diamine	7.36	32,593,173.1
97-39-2	1,2-bis(2-methylphenyl) guanidine	8.52	59,877,027.0
127-63-9	Benzenesulfonylbenzene	10.96	5,598,634.0
3622-84-2	*N*-butylbenzenesulfonamide	11.38	1,760,919.5
109-43-3	dibutyl decanedioate	16.67	31,284,068.5
301-02-0	(*Z*)-octadec-9-enamide	17.1	7,128,308.5
103-23-1	bis(2-ethylhexyl) hexanedioate	17.71	27,337,269.8
78-33-1	tris(4-tert-butylphenyl) phosphate	17.81	129,976,466.5
540-10-3	Hexadecyl hexadecanoate	17.78	468,840.4
2403-88-5	2,2,6,6-tetramethylpiperidin-4-ol	1.08	19,585,172.7
80-09-1	4-(4-hydroxyphenyl) sulfanylphenol	8.65	3,766,994.7
78-40-0	Triethyl phosphate	9.49	23,399,462.7
778-28-9	butyl 4-methylbenzenesulfonate	10.45	24,345.5
134-62-3	*N*,*N*-diethyl-3-methylbenzamide	11.74	36,836,082.3
4986-89-4	[2-(hydroxymethyl)-3-prop-2-enoyloxy-2-(prop-2-enoyloxymethyl)propyl] prop-2-enoate	12.21	6,454,678.4
80-18-2	methyl benzenesulfonate	13.07	64,883.4
71360-06-0	bis(3,5-dimethylphenyl) phosphane	13.7	127,148,710.4
124-30-1	octadecan-1-amine	16.64	63,886,631.2
629-54-9	Hexadecanamide	16.84	24,642,537.3
121-44-8	*N*,*N*-diethylethanamine	1.16	6,841,673.3
502-44-3	oxepan-2-one	4.96	1,851,724.8
149-30-4	3*H*-1,3-benzothiazole-2-thione	9.68	1,344,481.4
629-01-6	Octanamide	11.43	5,178,127.2
2358-84-1	2-[2-(2-methylprop-2-enoyloxy) ethoxy] ethyl 2-methylprop-2-enoate	11.94	6,987,279.9
1541-67-9	2-[dodecyl(2-hydroxyethyl) amino] ethanol	13.89	132,161,614.0
115-86-6	Triphenyl Phosphate	14.33	126,329,399.2
1620-98-0	3,5-di-tert-butyl-4-hydroxybenzaldehyde	14.43	10,122,291.2
3658-48-8	bis(2-ethylhexoxy)-oxophosphanium	15.04	8551.6
78-51-3	Tris(2-butoxyethyl) phosphate	15.42	98,345,028.7
78-30-8	Tris(2-methylphenyl) phosphate	15.70	143,057,783.9
32509-66-3	2-[3,3-bis(3-tert-butyl-4-hydroxyphenyl) butanoyloxy] ethyl 3,3-bis(3-tert-butyl-4-hydroxyphenyl) butanoate	16.88	17,810,936.6
112-84-5	(*Z*)-docos-13-enamide	17.96	6,052,480.5

**Table 5:** Data pertaining to the chromatographic retention of species evaluated under negative ionization, along with response slope values from individual calibration curves.

CAS	Name	Retention Time (min)	Response Slope (Peak Area/ppm Concentration)
70-49-5	2-sulfanylbutanedioic acid	0.6	65,531.6
121-34-6	4-Hydroxy-3-methoxybenzoic acid,	1.14	265,404.7
50-84-0	2,4-Dichlorobenzoic acid	6.81	287,978.1
20434-05-3	Bis(4-methoxyphenyl) phosphinic acid	9.61	5,837,765.8
115-39-9	2,6-dibromo-4-[3-(3,5-dibromo-4-hydroxyphenyl)-1,1-dioxo-2,1lambda6-benzoxathiol-3-yl] phenol	11.59	585,533.2
88-26-6	2,6-ditert-butyl-4-(hydroxymethyl) phenol	14.15	2,062,965.5
506-13-8	16-Hydroxy-hexadecanoic acid	15.44	3,415,425.7
514-10-3	(1R,4aR,4bR,10aR)-1,4a-dimethyl-7-propan-2-yl-2,3,4,4b,5,6,10,10a-octahydrophenanthrene-1-carboxylic acid	17.1	2,330,407.5
57-10-3	hexadecanoic acid	17.46	1,177,771.3
506-30-9	icosanoic acid	18.45	4,163,768.5
6683-19-8	[3-[3-(3,5-ditert-butyl-4-hydroxyphenyl) propanoyloxy]-2,2-bis [3-(3,5-ditert-butyl-4-hydroxyphenyl) propanoyloxymethyl] propyl] 3-(3,5-ditert-butyl-4-hydroxyphenyl) propanoate	19.31	2,763,898.8
100-21-0	Terephthalic Acid	0.6	465,975.7
90-64-2	2-hydroxy-2-phenylacetic acid	1.36	1,015,852.8
149-57-5	2-Ethylhexanoic acid	11.41	845,491.4
90-43-7	2-Phenylphenol	12.7	165,700.2
4376-20-9	2-(2-ethylhexoxycarbonyl) benzoic acid	13.82	3,050,868.3
20170-32-5	3-(3,5-Di-tert-butyl-4-hydroxyphenyl) propionic acid	13.94	3,376,097.3
128-37-0	2,6-ditert-butyl-4-methylphenol	16.41	232,745.4
36443-68-2	2-[2-[2-[3-(3-tert-butyl-4-hydroxy-5-methylphenyl) propanoyloxy] ethoxy] ethoxy] ethyl 3-(3-tert-butyl-4-hydroxy-5-methylphenyl) propanoate	16.25	5,584,911.8
88-24-4	2-tert-butyl-6-[(3-tert-butyl-5-ethyl-2-hydroxyphenyl) methyl]-4-ethylphenol	17.61	18,395,081.3
57-11-4	octadecanoic acid	17.98	2,294,766.8
1709-70-2	4-[[3,5-bis[(3,5-ditert-butyl-4-hydroxyphenyl) methyl]-2,4,6-trimethylphenyl] methyl]-2,6-ditert-butylphenol	19.56	3,527,892.8

**Table 6:** Data pertaining to the chromatographic retention of species evaluated using GC-MS, along with response slope values from individual calibration curves.

CAS	IUPAC Name	Retention Time (min)	Response Slope (Peak Area/ppm Concentration)
541-05-9	2,2,4,4,6,6-hexamethyl-1,3,5,2,4,6-trioxatrisilinane	4.3	914,666.8
123-05-7	2-ethylhexanal	6.4	842,858.9
100-52-7	benzaldehyde	6.5	341,982.6
111-13-7	octan-2-one	7.0	816,866.4
556-67-2	2,2,4,4,6,6,8,8-octamethyl-1,3,5,7,2,4,6,8-tetraoxatetrasilocane	7.1	1,062,178.4
104-76-7	2-ethylhexan-1-ol	7.6	550,482.7
6294-40-2	1-bromo-4-methylcyclohexane	7.7	567,268.8
1678-93-9	butylcyclohexane	7.7	362,041.2
823-76-7	1-cyclohexylethanone	7.7	37,097.9
98-86-2	1-phenylethanone	8.3	881,780.5
617-94-7	2-phenylpropan-2-ol	8.6	691,246.1
122-00-9	1-(4-methylphenyl) ethanone	10.1	403,324.6
1126-79-0	butoxybenzene	10.1	821,422.5
112-41-4	dodec-1-ene	10.2	313,423.4
7169-34-8	1-benzofuran-3-one	10.5	54,448.2
1731-84-6	methyl nonanoate	10.7	522,552.7
103-11-7	2-ethylhexyl prop-2-enoate	10.7	380,414.3
7473-98-5	2-hydroxy-2-methyl-1-phenylpropan-1-one	11.5	360,269.4
148-53-8	2-hydroxy-3-methoxybenzaldehyde	11.8	83,272.7
112-29-8	1-bromodecane	12.3	142,018.3
608-27-5	2,3-dichloroaniline	12.3	237,885.3
321-60-8	1-fluoro-2-phenylbenzene	12.5	599,634.6
141-28-6	diethyl hexanedioate	12.6	261,867.7
1120-36-1	tetradec-1-ene	12.7	330,158.3
719-22-2	2,6-ditert-butylcyclohexa-2,5-diene-1,4-dione	13.6	103,298.9
7283-69-4	bis(2-methylpropyl) (*E*)-but-2-enedioate	13.7	314,769.3
96-76-4	2,4-ditert-butylphenol	13.9	576,435.9
128-37-0	2,6-ditert-butyl-4-methylphenol	14.0	522,447.2
2162-98-3	1,10-dichlorodecane	14.3	732,021.1
544-76-3	hexadecane	14.7	382,995.0
119-61-9	diphenylmethanone	15.0	490,375.2
636-09-9	diethyl benzene-1,4-dicarboxylate	15.2	332,441.9
131-58-8	(2-methylphenyl)-phenylmethanone	15.3	349,309.7
451-40-1	1,2-diphenylethanone	15.8	814,712.9
1620-98-0	3,5-ditert-butyl-4-hydroxybenzaldehyde	16.1	376,847.4
84-74-2	dibutyl benzene-1,2-dicarboxylate	17.4	866,586.2
301-02-0	(*Z*)-octadec-9-enamide	19.9	100,949.6
115-86-6	triphenyl phosphate	20.3	147,008.6
88-24-4	2-tert-butyl-6-[(3-tert-butyl-5-ethyl-2-hydroxyphenyl) methyl]-4-ethylphenol	21.1	207,657.5
117-81-7	bis(2-ethylhexyl) benzene-1,2-dicarboxylate	21.3	694,013.2
112-84-5	(*Z*)-docos-13-enamide	22.9	214,488.5
111-02-4	(6E,10E,14E,18E)-2,6,10,15,19,23-hexamethyltetracosa-2,6,10,14,18,22-hexaene	23.2	442,697.1

**Table 7:** Allocation of substances based on the quantitation of pentaerythritol tetraacrylate, the substance proposed for use as an internal standard.

Categories	In the Critical Population	Total Population	Considering the 0.5 UF Value	
**True Positive**	16/21	16/26	20/21	20/26
**False Negative**	5/21	10/26	1/21	6/26
**Sensitivity**	76.2%	61.5% *	**95.2%**	**76.9%**

* 100% error in the classification of sulfur-polarizable bond-containing species.

**Table 8:** Allocation of substances based on the quantitation of bromophenol blue, the substance proposed for use as an internal standard.

Categories	Total Population	Considering the 0.5 UF Value
**True Positive**	16/22	19/22
**False Negative**	6/22	3/22
**Sensitivity**	72.7%	**86.4%**

**Table 9:** Allocation of substances based on the quantitation of dodec-1-ene and 1-fluoro-2-phenylbenzene, the substances proposed for use as internal standards.

Selected IS	Dodec-1-Ene (CAS: 112-41-4)		1-Fluoro-2-Phenylbenzene (CAS: 321-60-8)	
**Scenarios**	Non-Accounting for UF	With UF	Non-Accounting for UF	With UF
**True Positive**	35/42	35/42	11/42	31/42
**False Negative**	7/42	7/42	31/42	11/42
**Sensitivity**	71.4%	83.3%	26.2%	73.8%

## Data Availability

Data presented in this study are available on request from the corresponding authors.

## Supplementary Materials

The following supporting information can be downloaded at: https://www.mdpi.com/article/10.3390/molecules28155772/s1, Table S1: Amines and amides, Table S2: Carboxylic acids, Table S3: Phenols, Table S4: Polarized oxygen bonds, Table S5: Sulfur-polarized bonds, Table S6: Phosphorus-polarized bonds, Table S7: Splitting the compounds of the LC-MS-based process into sets. The prime (in terms of abundance) ion for the detection is also noted. Unless otherwise specified, the ion corresponds to the protonated substance [M + H]^+^ in positive ionization and the deprotonated substance [M-H]^-^ in negative ionization, Table S8: The analytes used in response factor generation for GC-MS, Table S9: Response slopes for the different analytes evaluated under positive ionization: Q1, median, Q3 descriptive statistic values for the values attained in variation experiments and the %RSD, Table S10: Response slopes for the different analytes evaluated under negative ionization: Q1, median, Q3 descriptive statistic values for the values attained in variation experiments and the %RSD.
