# Supplementary material for: Determination of Response Factors for Analytes Detected during Migration Studies, Strategy and Internal Standard Selection for Risk Minimization
Source: Molecules. 2023 Jul 31;28(15):5772. doi: 10.3390/molecules28155772 (PMC10421053; doi:10.3390/molecules28155772)
Supplement: Supplementary file 1 [file molecules-28-05772-s001.zip › molecules-2437177-supplementary.pdf]

Article

Determination of Response Factors for Analytes detected during Migration Studies, Strategy and Internal Standard Selection for Risk Minimization

Nikolaos Kritikos, Anna Bletsou, Christina Konstantinou, Antonios - Dionysios Neofotistos, Constantinos Kousoulos and Yannis Dotsikas

SUPPLEMENTARY MATERIAL

Table S1. Amines & amides

No.	IUPAC Nomenclature/ CAS	Molecular Formula	LogP	PSA (Å²)	Polarizability (Å³)	pKa*
**1**	1-[bis(2-hydroxypropyl) amino] propan-2-ol, CAS: 122-20-3	$C_9H_{21}NO_3$	-0.07	63.9	20.7	14.8 (9.3)
**2**	2,2,6,6-Tetramethyl-piperidin-4-ol, CAS: 2403-88-5	$C_9H_{19}NO$	0.52	32.3	18.7	15.1(10.1)
**3**	5-[(3,4,5-trimethoxyphenyl) methyl] pyrimidine-2,4-diamine, CAS: 738-70-5	$C_{14}H_{18}N_4O_3$	1.28	105.5	29.8	(7.2)
**4**	N,N-diethyl-3-methylbenzamide, CAS: 134-62-3	$C_{12}H_{17}NO$	2.50	20.3	22.3	(-1.0)
**5**	Octanamide, CAS: 629-01-6	$C_8H_{17}NO$	1.89	43.1	16.7	(-1.4)
**6**	N,N-diethylethanamine, CAS: 121-44-8	$C_6H_{15}N$	1.26	3.2	13.3	(10.2)
**7**	N-butylbutan-1-amine, CAS: 111-92-2	$C_8H_{19}N$	2.46	12.0	17.0	(10.8)
**8**	1,2-bis(2-methylphenyl) guanidine, CAS: 97-39-2	$C_{15}H_{17}N_3$	4.16	47.9	28.3	(9.4)
**9**	2-[dodecyl(2-hydroxyethyl) amino] ethanol, CAS: 1541-67-9	$C_{16}H_{35}NO_2$	3.21	49.3	28.3	-1.3 (15.6)
**10**	Hexadecanamide, CAS: 629-54-9	$C_{16}H_{33}NO$	5.45	43.1	31.4	(-1.4)
**11**	(Z)-octadec-9-enamide, CAS: 301-02-0	$C_{18}H_{35}NO$	5.98	43.1	34.9	(-1.4)
**12**	octadecan-1-amine, CAS: 124-30-1	$C_{18}H_{39}N$	6.92	26.0	35.5	(10.2)
**13**	(Z)-docos-13-enamide, CAS: 112-84-5	$C_{22}H_{43}NO$	7.76	43.1	42.3	(-1.4)

* *Presented as the strongest acidic pKa, with the respective values for the strongest basic pKa provided in parentheses*

Table S2. Carboxylic acids

No.	Compound	Molecular Formula	LogP	PSA (Å²)	Polarizability (Å³)	pKa*
**1**	2-sulfanylbutanedioic acid, CAS: 70-49-5	$C_4H_6O_4S$	-0.07	74.6	12.4	3.7
**2**	Terephthalic acid, CAS: 100-21-0	$C_8H_6O_4$	1.29	74.6	15.1	3.3
**3**	2,4-Dichlorobenzoic acid, CAS: 50-84-0	$C_7H_4Cl_2O_2$	2.84	37.3	16.6	2.9
**4**	2-(2-ethylhexoxycarbonyl) benzoic acid, CAS: 4376-20-9	$C_{16}H_{22}O_4$	4.66	63.6	30.0	3.1
**5**	16-Hydroxy-hexadecanoic acid, CAS: 506-13-8	$C_{16}H_{32}O_3$	4.82	57.5	31.3	5.0(-2.0)
**6**	4-Hydroxy-3-methoxybenzoic acid, CAS: 121-34-6	$C_8H_8O_4$	1.17	66.8	15.9	4.2
**7**	hexadecanoic acid, CAS: 57-10-3	$C_{16}H_{32}O_2$	6.26	37.3	30.7	5.0
**8**	octadecanoic acid, CAS: 57-11-4	$C_{18}H_{36}O_2$	7.15	37.3	34.4	5.0
**9**	icosanoic acid, CAS: 506-30-9	$C_{20}H_{40}O_2$	8.04	37.3	38.1	5.0
**10**	3-(3,5-Di-tert-butyl-4-hydroxyphenyl) propionic acid, CAS: 20170-32-5	$C_{17}H_{26}O_3$	4.84	57.5	31.5	4.8
**11**	2-ethylhexanoic acid, CAS: 149-57-5	$C_8H_{16}O_2$	2.80	37.3	16.0	5.1
**12**	2-hydroxy-2-phenylacetic acid, CAS: 90-64-2	$C_8H_8O_3$	0.90	57.5	15.2	3.8
**13**	(1R,4aR,4bR,10aR)-1,4a-dimethyl-7-propan-2-yl-2,3,4,4b,5,6,10,10a-octahydrophenanthrene-1-carboxylic acid, CAS: 514-10-3	$C_{20}H_{30}O_2$	4.95	37.3	35.4	4.6

* *Presented as the strongest acidic pKa, with the respective values for the strongest basic pKa provided in parentheses*

Table S3. Phenols

No.	Compound	Molecular Formula	LogP	PSA (Å²)	Polarizability (Å³)	pKa*
**1**	4-(4-hydroxyphenyl)sulfanylphenol, CAS: 80-09-1	$C_{12}H_{10}O_4S$	2.32	74.6	25.6	7.4
**2**	2-Phenylphenol, CAS: 90-43-7	$C_{12}H_{10}O$	3.32	20.2	22.0	9.7
**3**	2,6-ditert-butyl-4-(hydroxymethyl)phenol, CAS: 88-26-6	$C_{15}H_{24}O_2$	3.99	40.5	28.0	10.7
**4**	2,6-dibromo-4-[3-(3,5-dibromo-4-hydroxyphenyl)-1,1-dioxo-2,1lambda6-benzoxathiol-3-yl] phenol, CAS: 115-39-9	$C_{19}H_{10}Br_4O_5S$	7.18	83.8	48.6	6.4
**5**	2,6-ditert-butyl-4-methylphenol, CAS: 128-37-0	$C_{15}H_{24}O$	5.27	20.2	27.3	11.6
**6**	2-[2-[2-[3-(3-tert-butyl-4-hydroxy-5-methylphenyl) propanoyloxy] ethoxy] ethoxy] ethyl 3-(3-tert-butyl-4-hydroxy-5-methylphenyl) propanoate, CAS: 36443-68-2	$C_{34}H_{50}O_8$	7.65	111.5	64.3	10.2
**7**	2-tert-butyl-6-[(3-tert-butyl-5-ethyl-2-hydroxyphenyl) methyl]-4-ethylphenol, CAS: 88-24-4	$C_{25}H_{36}O_2$	8.46	40.5	44.7	8.9
**8**	4-[[3,5-bis[(3,5-ditert-butyl-4-hydroxyphenyl) methyl]-2,4,6-trimethylphenyl] methyl]-2,6-ditert-butylphenol, CAS: 1709-70-2	$C_{54}H_{78}O_3$	18.15	60.7	95.4	10.9
**9**	[3-[3-(3,5-ditert-butyl-4-hydroxyphenyl) propanoyloxy]-2,2-bis[3-(3,5-ditert-butyl-4-hydroxyphenyl) propanoyloxymethyl] propyl] 3-(3,5-ditert-butyl-4-hydroxyphenyl) propanoate, CAS: 6683-19-8	$C_{73}H_{108}O_{12}$	19.29	186.1	134.2	10.2

* *Presented as the strongest acidic pKa, with the respective values for the strongest basic pKa provided in parentheses*

Table S4. Polarized oxygen bonds

No.	Compound	Molecular Formula	LogP	PSA (Å²)	Polarizability (Å³)	pKa*
**1**	oxepan-2-one, CAS: 502-44-3	$C_6H_{10}O_2$	1.04	26.3	11.8	n/a**
**2**	[3-prop-2-enoyloxy-2,2-bis(prop-2-enoyloxymethyl) propyl] prop-2-enoate, CAS: 4986-89-4	$C_{17}H_{20}O_8$	2.98	105.2	34.3	n/a
**3**	dibutyl decanedioate, CAS: 109-43-3	$C_{18}H_{34}O_4$	5.21	52.6	35.4	n/a
**4**	bis(2-ethylhexyl) hexanedioate, CAS: 103-23-1	$C_{22}H_{42}O_4$	6.83	52.6	42.8	n/a
**5**	Hexadecyl hexadecanoate, CAS: 540-10-3	$C_{32}H_{64}O_2$	13.06	26.3	60.5	n/a
**6**	2-[3,3-bis(3-tert-butyl-4-hydroxyphenyl) butanoyloxy] ethyl 3,3-bis(3-tert-butyl-4-hydroxyphenyl) butanoate, CAS: 32509-66-3	$C_{50}H_{66}O_8$	12.64	133.5	90.6	9.5
**7**	3,5-Di-tert-butyl-4-hydroxybenzaldehyde, CAS: 1620-98-0	$C_{15}H_{22}O_2$	4.47	37.3	27.4	8.6
**8**	2-[2-(2-methylprop-2-enoyloxy) ethoxy] ethyl 2-methylprop-2-enoate, CAS: 2358-84-1	$C_{12}H_{18}O_5$	2.40	61.8	24.6	n/a

* *Presented as the strongest acidic pKa, with the respective values for the strongest basic pKa provided in parentheses*

** *n/a: not applicable*

Table S5. Sulfur polarized bonds

No.	Compound	Molecular Formula	LogP	PSA (Å²)	Polarizability (Å³)	pKa*
**1**	Methyl benzenesulfonate, CAS: 80-18-2	$C_7H_8O_3S$	1.53	43.4	17.0	n/a**
**2**	3H-1,3-benzothiazole-2-thione, CAS: 149-30-4	$C_7H_5NS_2$	2.89	12.9	18.7	3.6
**3**	Benzenesulfonylbenzene, CAS: 127-63-9	$C_{12}H_{10}O_2S$	2.93	34.1	24.4	n/a
**4**	butyl 4-methylbenzenesulfonate, CAS: 778-28-9	$C_{11}H_{16}O_3S$	3.37	43.4	24.3	n/a
**5**	2-methylbenzenesulfonamide, CAS: 88-19-7	$C_7H_9NO_2S$	1.09	60.2	17.4	10.4
**6**	N-butylbenzenesulfonamide, CAS: 3622-84-2	$C_{10}H_{15}NO_2S$	2.13	46.2	23.0	10.2

** Presented as the strongest acidic pKa, with the respective values for the strongest basic pKa provided in parentheses*

*** n/a: not applicable*

Table S6. Phosphorus polarized bonds

No.	Compound	Molecular Formula	LogP	PSA (Å²)	Polarizability (Å³)	pKa*
**1**	Triethyl phosphate, CAS: 78-40-0	$C_6H_{15}O_4P$	1.18	44.8	17.3	n/a**
**2**	Tris(2-butoxyethyl) phosphate, CAS: 78-51-3	$C_{18}H_{39}O_7P$	3.95	72.5	41.4	n/a
**3**	Tris(4-tert-butylphenyl) phosphate, CAS: 78-33-1	$C_{30}H_{39}O_4P$	9.72	44.8	56.7	n/a
**4**	Triphenyl phosphate, CAS: 115-86-6	$C_{18}H_{15}O_4P$	5.09	44.8	34.8	n/a
**5**	tris(2-methylphenyl) phosphate, CAS: 78-30-8	$C_{21}H_{21}O_4P$	6.63	44.8	40.1	n/a
**6**	bis(2-ethylhexoxy)-oxophosphanium, CAS: 3658-48-8	$C_{16}H_{34}O_3P^+$	5.78	41.5	34.6	15.3(-0.9)
**7**	bis(3,5-dimethylphenyl) phosphane, CAS: 71360-06-0	$C_{16}H_{19}P$	5.05	0.0	29.8	n/a
**8**	bis(4-methoxyphenyl) phosphinic acid, CAS: 20434-05-3	$C_{14}H_{15}O_4P$	2.11	55.8	28.6	2.4

** Presented as the strongest acidic pKa, with the respective values for the strongest basic pKa provided in parentheses*

*** n/a: not applicable*

Table S7. The split of the compounds of the LC-MS based process into sets. The prime (in terms of abundance) ion for the detection is also noted. Unless otherwise specified, the ion corresponds to the protonated substance $[M+H]^+$ in positive ionization and the deprotonated substance $[M-H]^-$ in negative ionization.

ESI (+)			
**Set 1 (12 compounds)**		**Set 2 (10 compounds)**	
**Analyte (IUPAC Name/CAS)**	**Prime Ion *m/z***	**Analyte (IUPAC Name/CAS)**	**Prime Ion *m/z***
1-[bis(2-hydroxypropyl) amino] propan-2-ol, CAS: 122-20-3	192.1600	2,2,6,6-tetramethylpiperidin-4-ol, CAS: 2403-88-5	158.1545
2-methylbenzenesulfonamide, CAS: 88-19-7	194.0252 $[Na^+]$	Triethyl phosphate, CAS: 78-40-0	183.0786
5-[(3,4,5-trimethoxyphenyl) methyl] pyrimidine-2,4-diamine, CAS: 738-70-5	291.1457	Methyl benzenesulfonate, CAS: 80-18-2	173.0272
N-butylbenzenesulfonamide, CAS: 3622-84-2	214.0902	4-(4-hydroxyphenyl) sulfanylphenol, CAS: 80-09-1	251.0378
N-butylbutan-1-amine, CAS: 111-92-2	130.1596	N,N-diethyl-3-methylbenzamide, CAS: 134-62-3	192.1388
Benzenesulfonylbenzene, CAS: 127-63-9	219.0480	[2-(hydroxymethyl)-3-prop-2-enoyloxy-2-(prop-2-enoyloxymethyl)propyl] prop-2-enoate, CAS: 4986-89-4	370.1502
(Z)-octadec-9-enamide, CAS: 301-02-0	282.2797	butyl 4-methylbenzenesulfonate, CAS: 778-28-9	229.0898
1,2-bis(2-methylphenyl) guanidine, CAS: 97-39-2	240.1501	bis(3,5-dimethylphenyl) phosphane, CAS: 71360-06-0	260.1568 $[NH_4^+]$
dibutyl decanedioate, CAS: 109-43-3	315.2535	Hexadecanamide, CAS: 629-54-9	256.2640
bis(2-ethylhexyl) hexanedioate, CAS: 103-23-1	388.3427 $[NH_4^+]$	octadecan-1-amine, CAS: 124-30-1	270.3161
tris(4-tert-butylphenyl) phosphate, CAS: 78-33-1	495.2664		
Hexadecyl hexadecanoate, CAS: 540-10-3	498.5250 $[NH_4^+]$		

Set 3 (13 compounds)	
**Analyte (IUPAC Name/ CAS)**	**Prime Ion *m/z***
oxepan-2-one, CAS: 502-44-3	115.0759
N,N-diethylethanamine, CAS: 121-44-8	102.1283
Octanamide, CAS: 629-01-6	144.1388
2-[2-(2-methylprop-2-enoyloxy) ethoxy] ethyl 2-methylprop-2-enoate, CAS: 2358-84-1	243.1232
3H-1,3-benzothiazole-2-thione, CAS: 149-30-2	167.9942
2-[dodecyl(2-hydroxyethyl) amino] ethanol, CAS: 1541-67-9	274.2746
Tris(2-butoxyethyl) phosphate, CAS: 78-51-3	399.2512
3,5-Di-tert-butyl-4-hydroxybenzaldehyde, CAS: 1620-98-0	235.1698
Triphenyl phosphate, CAS: 115-86-6	327.0786
bis(2-ethylhexoxy)-oxophosphanium, CAS: 3658-48-8	305.2246
tris(2-methylphenyl) phosphate, CAS 78-30-8	369.1256
(Z)-docos-13-enamide, CAS: 112-84-5	338.3423
2-[3,3-bis(3-tert-butyl-4-hydroxyphenyl) butanoyloxy] ethyl 3,3-bis(3-tert-butyl-4-hydroxyphenyl) butanoate, CAS: 32509-66-3	812.5101 [NH_4^+]

ESI (-)			
**Set 4 (11 compounds)**		**Set 5 (11 compounds)**	
**Analyte (IUPAC Name/ CAS)**	**Prime Ion *m/z***	**Analyte (IUPAC Name/ CAS)**	**Prime Ion *m/z***
2-sulfanylbutanedioic acid, CAS: 70-49-5	148.9909	2-hydroxy-2-phenylacetic acid, CAS: 90-64-2	151.0395
4-Hydroxy-3-methoxybenzoic acid, CAS: 121-34-6	167.0344	Terephthalic acid, CAS: 100-21-0	165.0188
Bis(4-methoxyphenyl) phosphinic acid, CAS: 20434-05-3	277.0630	2-Ethylhexanoic acid, CAS: 149-57-5	143.1072
2,4-Dichlorobenzoic acid, CAS: 50-84-0	188.9510	2-Phenylphenol, CAS: 90-43-7	169.0653

2,6-ditert-butyl-4-(hydroxymethyl) phenol, CAS: 88-26-6	235.1690	2-(2-ethylhexoxycarbonyl) benzoic acid, CAS: 4376-20-9	277.1440
16-Hydroxyhexadecanoic acid, CAS: 506-13-8	271.2273	3-(3,5-Di-tert-butyl-4-hydroxyphenyl) propionic acid, CAS: 20170-32-5	277.1804
(1R,4aR,4bR,10aR)-1,4a-dimethyl-7-propan-2-yl-2,3,4,4b,5,6,10,10a-octahydrophenanthrene-1-carboxylic acid, CAS: 514-10-3	301.2168	2,6-ditert-butyl-4-methylphenol, CAS: 128-37-0	219.1749
hexadecanoic acid, CAS: 57-10-3	255.2324	octadecanoic acid, CAS: 57-11-4	283.2637
2,6-dibromo-4-[3-(3,5-dibromo-4-hydroxyphenyl)-1,1-dioxo-2,1lambda6-benzoxathiol-3-yl] phenol, CAS: 115-39-9	664.6904	2-[2-[2-[3-(3-tert-butyl-4-hydroxy-5-methylphenyl) propanoyloxy] ethoxy] ethoxy] ethyl 3-(3-tert-butyl-4-hydroxy-5-methylphenyl) propanoate, CAS: 36443-68-2	585.3427
icosanoic acid, CAS: 506-30-9	311.2950	2-tert-butyl-6-[(3-tert-butyl-5-ethyl-2-hydroxyphenyl) methyl]-4-ethylphenol, CAS: 88-24-4	367.2637
[3-[3-(3,5-ditert-butyl-4-hydroxyphenyl) propanoyloxy]-2,2-bis[3-(3,5-ditert-butyl-4-hydroxyphenyl) propanoyloxymethyl] propyl] 3-(3,5-ditert-butyl-4-hydroxyphenyl) propanoate, CAS: 6683-19-8	1175.7763	4-[[3,5-bis[(3,5-ditert-butyl-4-hydroxyphenyl) methyl]-2,4,6-trimethylphenyl] methyl]-2,6-ditert-butylphenol, CAS: 1709-70-2	773.5873

Table S8. The analytes used in the response factor generation for GC-MS.

Set	IUPAC Name/ CAS		*m/z*	BP (°C) / logP
1 (15 species)	2,2,4,4,6,6-hexamethyl-1,3,5,2,4,6-trioxatrisilinane	541-05-9	207	134.0/ 0.58
	octan-2-one	111-13-7	58	172.5/ 2.59
	benzaldehyde	100-52-7	51	179.0/ 1.38
	2-ethylhexan-1-ol	104-76-7	57	184.6/ 2.50
	butoxybenzene	1126-79-0	94	210.0/ 3.14
	dodec-1-ene	112-41-4	56	213.8/ 5.50
	tetradec-1-ene	1120-36-1	69	233.0/ 6.38
	1-fluoro-2-phenylbenzene	321-60-8	172	248.0/ 3.76
	2-hydroxy-2-methyl-1-phenylpropan-1-one	7473-98-5	59	260.8/ 1.72
	2-hydroxy-3-methoxybenzaldehyde	148-53-8	152	265.5/ 1.88
	2,6-ditert-butylcyclohexa-2,5-diene-1,4-dione	719-22-2	177	285.4/ 3.88
	diethyl benzene-1,4-dicarboxylate	636-09-9	177	302.0/ 2.69
	1,2-diphenylethanone	451-40-1	105	320.0/ 3.37
	bis(2-ethylhexyl) benzene-1,2-dicarboxylate	117-81-7	149	384.0/ 8.03
	(6E,10E,14E,18E)-2,6,10,15,19,23-hexamethyltetracosa-2,6,10,14,18,22-hexaene	111-02-4	69	429.3/ 10.42
2 (14 species)	2-ethylhexanal	123-05-7	57	163.0/ 2.64
	1-bromo-4-methylcyclohexane	6294-40-2	97	174.6/ 3.11
	1-cyclohexylethanone	823-76-7	71	180.5/ 2.22
	methyl nonanoate	1731-84-6	74	213.5/ 3.29
	1-(4-methylphenyl)ethanone	122-00-9	119	226.0/ 2.04
	1-bromodecane	112-29-8	57	240.6/ 5.01
	1-benzofuran-3-one	7169-34-8	134	249.6/ 1.08
	2,4-ditert-butylphenol	96-76-4	191	263.5/ 4.76
	bis(2-methylpropyl) (E)-but-2-enedioate	7283-69-4	155	285.0/ 3.21
	Hexadecane	544-76-3	57	286.8/ 7.58
	diphenylmethanone	119-61-9	105	305.4/ 3.43
	dibutyl benzene-1,2-dicarboxylate	84-74-2	149	340.0/ 4.63
	(Z)-octadec-9-enamide	301-02-0	59	394.9/ 5.98
	2-tert-butyl-6-[(3-tert-butyl-5-ethyl-2-hydroxyphenyl)methyl]-4-ethylphenol	88-24-4	191	452.1/ 8.46
3 (13 species)	2,2,4,4,6,6,8,8-octamethyl-1,3,5,7,2,4,6,8-tetraoxatetrasilocane	556-67-2	281	175.8/ 0.77
	butylcyclohexane	1678-93-9	82	180.9/ 4.29
	1-phenylethanone	98-86-2	105	202.0/ 1.53
	2-ethylhexyl prop-2-enoate	103-11-7	55	213.5/ 3.94
	2-phenylpropan-1-ol	1123-85-9	121	232.2/ 1.86
	diethyl hexanedioate	141-28-6	157	245.0/ 1.50

2,3-dichloroaniline	608-27-5	161	252.0/ 2.35
2,6-ditert-butyl-4-methylphenol	128-37-0	205	265.0/ 5.27
1,10-dichlorodecane	2162-98-3	91	275.0/ 4.75
3,5-ditert-butyl-4-hydroxybenzaldehyde	1620-98-0	219	289.2/ 4.47
(2-methylphenyl)-phenylmethanone	131-58-8	195	308.0/ 3.95
triphenyl phosphate	115-86-6	326	370.0/ 5.09
(Z)-docos-13-enamide	112-84-5	59	474.2/ 7.76

Table S9. Response slopes for the different analytes evaluated under positive ionization – Q_1, median, Q_3 descriptive statistic values for the values attained in variation experiments and the %RSD

IUPAC Name	Q_1	Median	Q_3	%RSD
1-[bis(2-hydroxypropyl)amino] propan-2-ol	23783842	29601810	36150226	24.0
N-butylbutan-1-amine	421238	579641	664805	23.3
2-methylbenzenesulfonamide	5366	24804	77988	97.9
5-[(3,4,5-trimethoxyphenyl) methyl] pyrimidine-2,4-diamine	33324369	42001608	47399222	17.5
1,2-bis(2-methylphenyl)guanidine	50421441	59877027	75581978	22.4
benzenesulfonylbenzene	947468	5598634	9940923	85.8
N-butylbenzenesulfonamide	263955	1760920	3700566	94.5
dibutyl decanedioate	26165966	38439203	48810881	31.6
(Z)-octadec-9-enamide	4749471	10452554	22732456	78.6
bis(2-ethylhexyl) hexanedioate	23943937	27337270	59665371	54.0
tris(4-tert-butylphenyl) phosphate	105366035	129976467	201305495	35.1
hexadecyl hexadecanoate	281266	400002	456744	24.9
2,2,6,6-tetramethylpiperidin-4-ol	17352065	19585173	21539889	12.3
4-(4-hydroxyphenyl) sulfonylphenol	2761451	3766995	6132140	41.8
triethyl phosphate	21523113	23399463	29872415	17.1
butyl 4-methylbenzenesulfonate	10857	19939	29593	50.0
N,N-diethyl-3-methylbenzamide	23189851	31196421	42897496	36.1
[3-prop-2-enoyloxy-2,2-bis(prop-2-enoyloxymethyl)propyl] prop-2-enoate	1917471	5962548	8183457	65.1
methyl benzenesulfonate	4781	64883	376115	119.0
bis(3,5-dimethylphenyl)-oxophosphanium	94091322	127148710	158963866	26.4
octadecan-1-amine	56231448	63886631	100939640	31.1
hexadecanamide	15633137	24642537	44667660	65.0
N,N-diethylethanamine	7218416	7900134	9107039	14.2
oxepan-2-one	1291339	1851725	2805960	43.6
3H-1,3-benzothiazole-2-thione	850325	1344481	2170667	49.0
octanamide	3294418	5178127	9432607	62.0
2-[2-(2-methylprop-2-enoyloxy)ethoxy]ethyl 2-methylprop-2-enoate	5120374	6987280	9404664	29.9
2-[dodecyl(2-hydroxyethyl)amino]ethanol	100177102	132161614	165388137	24.7
triphenyl phosphate	90202616	126329399	150402618	28.4
3,5-ditert-butyl-4-hydroxybenzaldehyde	5243143	10122291	18821867	71.3
bis(2-ethylhexoxy)-oxophosphanium	4568	6810	9775	39.3
tris(2-butoxyethyl) phosphate	77215233	98345029	130449411	26.1
tris(2-methylphenyl) phosphate	106882676	143057784	178333205	25.8
Ethylene bis[3,3-bis(3-tert-butyl-4-hydroxyphenyl)butyrate]	14786139	17810937	26967918	30.9
(Z)-docos-13-enamide	864139	6052481	19483961	106.1

Table S10. Response slopes for the different analytes evaluated under negative ionization – Q_1, median, Q_3 descriptive statistic values for the values attained in variation experiments and the %RSD.

IUPAC Name	Q_1	Median	Q_3	%RSD
2-sulfanylbutanedioic acid	36250	65532	405475	116.6
4-hydroxy-3-methoxybenzoic acid	233546	265405	447964	37.0
2,4-dichlorobenzoic acid	266837	287978	355866	16.0
bis(4-methoxyphenyl) phosphinic acid	3572471	3996251	6043605	28.2
2,6-dibromo-4-[3-(3,5-dibromo-4-hydroxyphenyl)-1,1-dioxo-2,1 lambda 6-benzoxathiol-3-yl]phenol	509244	585533	726343	19.5
2,6-ditert-butyl-4-(hydroxymethyl)phenol	1858579	2062966	4125026	43.2
16-hydroxyhexadecanoic acid	3229104	3415426	5082677	25.3
(1R,4aR,4bR,10aR)-1,4a-dimethyl-7-propan-2-yl-2,3,4,4b,5,6,10,10a-octahydrophenanthrene-1-carboxylic acid	2858700	3601483	3856745	19.0
hexadecanoic acid	1044749	1572206	2851222	51.7
icosanoic acid	3434601	4065930	4197419	10.5
[3-[3-(3,5-ditert-butyl-4-hydroxyphenyl)propanoyloxy]-2,2-bis[3-(3,5-ditert-butyl-4-hydroxyphenyl) propanoyloxymethyl]propyl] 3-(3,5-ditert-butyl-4-hydroxyphenyl) propanoate	1983836	2626486	2754847	18.2
terephthalic acid	330053	481157	523527	32.0
2-hydroxy-2-phenylacetic acid	841876	1011499	1063310	14.2
2-ethylhexanoic acid	437559	845491	2096121	72.7
2-phenylphenol	91247	165700	422696	71.6
2-(2-ethylhexoxycarbonyl) benzoic acid	2714184	3050868	4352510	26.7
3-(3,5-ditert-butyl-4-hydroxyphenyl)propanoic acid	2958415	3376097	4588011	28.6
2,6-ditert-butyl-4-methylphenol	166065	232745	325335	32.9
2-[2-[2-[3-(3-tert-butyl-4-hydroxy-5-methylphenyl)propanoyloxy]ethoxy]ethoxy]ethyl 3-(3-tert-butyl-4-hydroxy-5-methylphenyl) propanoate	4636861	5584912	6315871	17.4
2-tert-butyl-6-[(3-tert-butyl-5-ethyl-2-hydroxyphenyl)methyl]-4-ethylphenol	15376289	17715272	19928074	15.1
octadecanoic acid	2256317	2339770	3494636	25.7
4-[[3,5-bis[(3,5-ditert-butyl-4-hydroxyphenyl)methyl]-2,4,6-trimethylphenyl]methyl]-2,6-ditert-butylphenol	3519332	3842086	5159569	22.3
